# Midwifery

**Published:** 1836-10

**Authors:** 


### MIDWIFERY.

*History of a successful Case of Cæsarean Section.* By Dr. Meyer, of Minden.

This is the fourth time Dr. Meyer has performed this operation, and three of his cases have terminated favorably. The subject of the following was the wife of a shoemaker, named Holle, residing at Minden, a woman of small size and slender make, aged thirty-eight, who had previously enjoyed good health, and borne three children easily and without any bad consequences. After this she was attacked with an arthritic affection, which, from poverty, privations, grief, and want of timely assistance, had increased to such a degree, that, for the space of a year, she only left her bed occasionally, and was then merely able to crawl about her room, bent double and holding by the chairs.

In this state she became again pregnant; an occurrence, of which the first intimation was given by the motions of the fœtus in utero. On the evening of the 19th of June labour came on, and towards morning the midwife in attendance, having discovered an abnormal state of the pelvis, advised her to have the assistance of an accoucheur. A neighbouring physician, Dr. Heilbronn, was called in, who, finding the pelvis so deformed as to render artificial delivery by dismemberment or the Cæsarean section unavoidable, ordered the patient to be blooded, and requested Dr. Meyer to take charge of the case.

An examination per vaginam showed that the pelvis was excessively deformed. On introducing the finger, which was done with difficulty, owing to a bending inwards of the ossa ilii and pubis, the summit of a round solid body was felt, which at first might be taken for the head of the child, but a more accurate examination proved it to be the distorted promontory of the sacrum, which projected close to the retreating symphysis pubis. Hence it was immediately concluded that the child lay entirely in the false pelvis; a circumstance which also explained the extreme prominence of the belly, which rested with the navel touching the external parts of generation.

Besides that the child was still living, the impossibility of getting at it through the vagina determined Dr. Meyer to abandon at once the idea of dismemberment, and he decided on this plan of operation, which the mother also pressed for; although, from her unfavorable condition, there was little hope of saving her. The further proceedings are thus described by Dr. Meyer:

"The patient was carried to the table, and placed on a straw mattress. The thighs could be separated only so far as merely to afford room for the hand of an examiner; the lumbar vertebræ appeared to be anchylosed with each other and with the sacrum, and it was impossible to place her in an horizontal posture. In this half-sitting posture, the abdomen presented a space of only about four inches from the umbilicus to the pubes. Notwithstanding this, and the presumption that the placenta lay in the line of the proposed incision, I made choice of it, for reasons which seemed to me conclusive.

"Two assistants stood, one on each side of the patient, with large soft sponges, warmed and slightly oiled; while Dr. Heilbronn stood at her feet, ready to hand the instruments and take hold of the child. The abdomen was then firmly and powerfully drawn up, and an incision, about four inches in length, was made through the integuments, commencing as close as possible to the umbilicus, and terminating at the pubes. The division of the linea alba and the peritoneum, to the same extent and in the same direction, gave exit to a considerable quantity of water, showing the coexistence of ascites. The discharge of this fluid, however, diminished the enormous tension of the abdomen, and contributed to facilitate the delivery. In all my former cases I had found the uterus extremely thin, scarcely thicker than stout paper, and easily divided: in the present instance, however, the walls were unusually firm and three lines in thickness. A new obstacle here arose: in cutting through the uterus, the knife entered the placenta, which was attached exactly opposite the line of incision. The consequences were, a division of the larger vessels which are collected about the centre of the placenta, giving rise to a considerable hemorrhage and a further interference with the previously limited space for the introduction of the hand and the passage of the child. No time, however, was to be lost: the section of the anterior wall of the uterus was speedily completed, the necessary detachment of the placenta was accomplished without any excessive hemorrhage, and the right knee of the child presented. As the opening in the womb was scarcely large enough for the passage of the head, it was necessary to extract the arms; after which the head followed, not without some resistance, yet without any laceration of the uterus. The extraction of the child was followed instantly by a remarkable contraction of the womb, and the bleeding stopped.

"Vomiting came on, accompanied by spasmodic contractions of the abdominal muscles, so that the assistants were obliged to direct their whole attention to the intestines, which were protruded with great force, and which were returned and kept in with much difficulty by the oiled sponges. When the blood and aqueous fluid were removed from the cavity of the abdomen, the uterus was seen contracted to about the size of two fists; and, as the somewhat swollen edges of the incision were tolerably approximated, the external wound was united by the bloody suture without further delay. After it was finished, a repetition of the vomiting forced a piece of omentum, about two inches long, through an interval between the sutures, two inches below the umbilicus: this was removed with the scissors without any hesitation. Immediately over the pubes an opening was left, about an inch in length, into which a tent of lint was inserted; compresses were placed at each side of the wound, a twelve-tailed bandage applied, and, in half an hour from the commencement of the operation, the patient, who during the whole time neither stirred nor uttered a single moan, was laid comfortably in bed, the vomiting had ceased, and the cough was less troublesome. The child, a male, come to its full time, and twenty inches in length, but imperfectly nourished, had breathed immediately after delivery, cried, and was doing well. About half an hour afterwards, during a violent paroxysm of coughing, a full half-ell of intestine was forced through the opening at the lower end of the wound, and lay between the thighs distended with flatus. Dr. Meyer, unwilling to undo the bandage in the absence of proper assistants, fortunately succeeded in reducing it by the taxis, and secured the opening with a compress and strips of adhesive plaster."

The patient ultimately got quite well.

From the successful termination of this and two other cases, Dr. Meyer is led to the conclusion that the Cæsarean section is much less dangerous than is generally supposed; that, when we have recourse to it in time and under favorable circumstances, it is oftener followed by a happy result than other violent modes of artificial delivery, adopted in case of great contraction and abnormal formation of the pelvis; and that, generally speaking, when there is a great probability of saving the mother, it should be preferred to dismembering the living child. Leaving the question undecided, whether the lateral incision should be selected because the section of the anterior wall of the uterus is likely to meet the placenta, his experience inclines him in all cases to make choice of the linea alba. Finally, he corrects a statement made by him in a paper inserted in Siebold's Journal, 3d band. 1st heft, that, "in such operations, only one intelligent assistant is necessary;" and declares it to be his conviction that three intelligent assistants are requisite,—two to prevent the protrusion of the intestines, and a third to remove the placenta and fœtus. *Neue Zeitschrift für Gebürtskunde.* Band. iii. Heft 1. 1835.

---

*Case of Extra-uterine Pregnancy.* By Dr. Löscher, of Lübben, and Professor Busch, of Berlin.

The wife of a taylor, at present in her thirtieth year, the mother of several children, ceased to menstruate in April, 1830. Other signs of pregnancy soon manifested themselves; viz. enlargement of the belly, changes in the breasts, and motions in the abdomen. These last were felt very strong in the sixteenth week, and were quite different from those of former pregnancies. Shortly after the twentieth week, she could distinguish accurately, in the left side of the belly, a round hard swelling, which she recognized as the head of a child; and from time to time individual parts of the child could be felt much more plainly than in former pregnancies. The motions, felt only at the upper circumference of the belly, which was distended on both sides, became gradually more painful, particularly at certain points, where the abdomen was so much protruded by its contents that she expected every moment the integuments would burst, and the child force its way through. The enlargement of the abdomen increased with surprising rapidity, so that, towards the end of the sixth month, it was as large as in the last month of ordinary pregnancy. The poor woman's condition was further aggravated by frequent attacks of colic and obstinate constipation yielding only to medicine, and by considerable swelling of the lower extremities. When at length the term of delivery approached, the belly, instead of sinking, remained full in the epigastric region; high up, the child could be felt lying transversely, and the patient had great distress of breathing. Slight pains, shooting from the spine to the thighs, indicated the commencement of labour; and a moderate discharge of blood rendered the presence of the district midwife desirable. Two entire days passed in this manner; the child moved briskly, but only in the upper part of the belly, and the pains became gradually weaker, when suddenly a large quantity of water (a bucketful, according to the patient's account,) was discharged from the vagina. The belly was thereby rendered much flatter, but the child still remained above in the same transverse position, and continued to move until the mother was seized with an universal rigor, when the pain entirely ceased, and the child gave no further signs of life. The midwife was again called, but could not be prevailed on to come: she assured them it was quite a mistake to suppose that labour had commenced, and that they must wait for stronger pains. Meanwhile, the favorable moment for saving the life of the child had passed by: it lay like a foreign body transversely in the umbilical region; milk flowed for several days from the breasts. After six weeks of distress, a copious flow of the menses appeared, (which continue still regular,) and her general health slowly improved; but she was weaker, had frequent attacks of syncope; the pains in the belly, which were generally moderate, became greatly aggravated before and during the menstrual period, and the tendency to constipation remained.

The belly sank by degrees; the child grew gradually smaller, harder to the touch, and as it were shrivelled up; it became also proportionably more moveable. During the course of five years the patient was examined by several physicians, who obtained a true knowledge of her condition, which she had long suspected; but she rejected every proposal of an operation, however easy of accomplishment and free from danger it might have been depicted to her. In fact, on several occasions, it appeared as if nature was about to form an abscess for the purpose of removing the child, which had now become as it were a foreign body; but the pains and other signs of inflammation in the vicinity of the umbilicus disappeared spontaneously. Nothing further was done than to apply a bandage to give adequate support to the abdomen, and to obviate the costiveness by the use of rhubarb and aloes.

When I examined her for the first time, on the 5th of May, in the sixth year of her pregnancy, her state of health was tolerably good. In addition to the pains before mentioned, her yellow clay-coloured hue, her sunken eyes, and her countenance, which wore a peculiar expression of suffering, betokened the existence of abdominal mischief. On making an examination per vaginam, which did not give the slightest pain, I found the uterus regularly formed, the cervix long and thick, the os uteri open, directed backwards, and deeper than usual. The belly was still remarkably swollen, almost as much as in the seventh month of a regular pregnancy: from the pubes to the navel it was so soft that I could pass my hand back almost to the spine without meeting the womb or any solid body; but, in the umbilical region, I could feel quite distinctly the child, hard and shrivelled, about twelve inches in length, lying transversely, the head bent on the breast, and the lower extremities turned towards the left side of the mother. I could also distinguish other parts of the child, as the elbows, shoulders, and ribs. It appeared to lie quite free in the cavity of the abdomen; for I could turn it in its long and transverse diameters, almost without giving the patient pain. Still, the usual position, before mentioned, is the most agreeable. Any slight rudiment of the placenta still remaining must be attached at the place where the child is connected to the mother by a string of withered funis. From the great mobility of the child, and from the possibility of grasping the abdominal integuments round it, and making them glide over it, as well as from the obstinate constipation, a portion of the small intestine would appear to be the spot where the fructified ovum grew, and derived its nutriment from the mother.

There can be no doubt that this is a case of extra-uterine and abdominal pregnancy, with which was combined, originally or subsequently, a dropsy of the uterus, although this organ is now free from every morbid affection. If the fœtus be not already a *lithopœdion*, it will assume that state by remaining longer in the abdomen. As it lies close behind the integuments of the belly, delivery by gastrotomy would not be liable to great difficulties, particularly as the linea alba offers the most favorable situation for the incision.

*Neue Zeitschrift für Geburtskunde.* Band iii. heft 2. 1835.

---

*Case of Fatal Hemorrhage from an Erectile Tumour of the Uterus.*
By Professor KILIAN, of Bonn.

A. B., twenty-four years old, strong and well-made, was admitted, in an advanced stage of pregnancy, into the Lying-in Hospital at Bonn. She was then extremely healthy, and did not remember to have ever suffered from illness. The menses made their first appearance in her fourteenth year, and afterwards regularly every three weeks. She had menstruated once during her pregnancy. At the expiration of her time she was delivered of a boy. Half an hour after delivery, the placenta came away, almost without assistance. At that time she was so well, that her medical attendant joked her on a presentiment of the fatal termination of her accouchement which she had frequently manifested, and which she, notwithstanding, still continued to nourish. The first three days subsequent to her delivery passed off without any symptom of disease. The secretions were healthy, and the general state of the patient highly satisfactory: but, during the afternoon of the fourth day, Dr. Kilian was sent for in great haste to see the patient, who was described to be swimming in her blood.

About two o'clock, whilst suckling her infant, she had exclaimed that she felt something boiling hot flowing between her legs, and immediately fainted. Before the nurse and house-surgeon could come to her assistance, the hemorrhage had ceased. When Dr. K. arrived, she had in a great measure recovered from the fainting fit into which the sudden loss of two and a half pounds of blood had thrown her. He proceeded to an examination, both externally and per vaginam. He found the uterus properly contracted, uniformly firm, and free from pain on pressure. The os uteri presented no coagulum, and was not more open than usual four days after delivery. Dr. K. was perfectly ignorant, and had indeed not the most distant suspicion, of the cause of the hemorrhage; for he had already carefully ascertained that the entire placenta had come away. Under these circumstances, all that could be prescribed were general preventive measures; such as the horizontal posture, repose, and cold applications to the external parts of generation, &c.

The patient continued gradually gathering strength till the 7th of February, when she was visited by a precisely similar attack. An examination was again made, but without any better result, and the former measures were continued. The patient recovered in a great measure her former strength; but, on the 13th of February, she was again surprised by a sudden hemorrhage. In all these attacks the blood uniformly ceased to flow before the nurse could reach the patient. This time the quantity lost amounted to about one pound and a half. Dr. Kilian was now in the greatest embarrassment. Having no knowledge of the essential nature of the disease, he was obliged to proceed empirically. He ordered bark, and the former measures to be continued.

On the 26th of February, the patient again lost two pounds of blood in a similar manner. Moreover, her subsequent prostration and debility were such as to leave no hope of her recovery. Opium was prescribed, and produced temporary relief; but the progress of the disease to a fatal termination was evidently not to be thwarted. The patient exhibited the greatest despondency, and kept continually sinking to the foot of the bed, notwithstanding the exertions of her attendants. The circulation was rapid, and the respiration correspondingly affected. Nevertheless, the patient's appetite continued tolerably good, and she was refreshed by sleep at intervals.

On the 3d of March, during the visit of the physician, the patient again exclaimed, in a weak voice, that she was bleeding: scarcely had she spoken when she was seized with convulsions, violent at first, and gradually weaker, amid which she expired.

At the post-mortem examination, all the viscera were found in a normal condition, except the uterus, and a cursory inspection of that did not betray its disease. It was pale, contracted, and firm; but on its front surface was observed a circular spot, rather larger than a half-crown piece, of a pale red colour, and less firm than the rest. An incision was made in the uterus posteriorly, and on its internal surface was discovered a tumour, corresponding to the above-mentioned spot, two inches long, and one and a half broad, of which the covering membrane hung down into the cavity of the uterus, and thus facilitated the inspection of its internal structure. This was extremely vascular: on looking into it, the open mouths of innumerable vessels were easily discernible by the naked eye. Around the tumour, the substance of the uterus was rather softer than elsewhere, and the numerous vessels leading towards it formed a concentric network.

A preparation has been made of this uterus, and it is now in the museum of the hospital.* *Hannoversche Annalen*, B. i. heft 1. 1836.

* A case resembling the above is related by Dr. Carswell in his tenth Fasciculus (Hemorrhage), and a beautiful representation of the tumour given in the fourth Plate (Fig. 2).—Eds.

